# The immunomodulatory effect of green tea (*Camellia sinensis*) leaves extract on immunocompromised Wistar rats infected by *Candida albicans*

**DOI:** 10.14202/vetworld.2018.765-770

**Published:** 2018-06-07

**Authors:** Retno P. Rahayu, Remita A. Prasetyo, Djoko A. Purwanto, Utari Kresnoadi, Regina P. D. Iskandar, Muhammad Rubianto

**Affiliations:** 1 Department of Oral and Maxillofacial Pathology, Faculty of Dental Medicine, Universitas Airlangga, Surabaya, Indonesia; 2 Installation of Oral and Dental Health, Dr. Soetomo Hospital, Surabaya, Indonesia; 3 Department of Pharmaceutical Chemistry, Faculty of Pharmacy, Universitas Airlangga, Surabaya, Indonesia; 4 Department of Prosthodontics, Faculty of Dental Medicine, Universitas Airlangga, Surabaya, Indonesia; 5 Student of Immunology, Postgraduate School, Universitas Airlangga, Surabaya, Indonesia; 6 Department of Periodontics, Faculty of Dental Medicine, Universitas Airlangga, Surabaya, Indonesia

**Keywords:** epigallocatechin gallate, epigallocatechin, green tea extract, immunocompromised, oral candidiasis

## Abstract

**Background and Aim:**

The immunocompromised condition is considered a defect in the immune system. This condition tends to increase the risk of oral candidiasis, due to the inability of the immune system to eliminate the adhesion of *Candida albicans* and leads to systemic candidiasis with a mortality rate of 60%. Green tea (*Camellia sinensis*) contains potential antioxidant and immunomodulatory which acts as anticancer, antifungal, and antivirus agent. The aim of this study was to invent herbal-based medicine, which acts as an immunomodulator and antifungal agent to treat fungal infection in immunocompromised patients.

**Materials and Methods:**

Thirty-five immunocompromised Wistar rats induced with *C. albicans* were divided into 7 groups (n=5): Control group (C+); treated for 4 days with green tea extract 1.25% (GT 4), epigallocatechin gallate (EGCG) 1% (EGCG 4), EGC 1% (EGC 4); and treated for 7 days with green tea extract 1.25% (GT 7), EGCG 1% (EGCG 7), and EGC 1% (EGC 7). Tongue tissue was collected and analyzed with immunohistochemistry staining using monoclonal antibody; interleukin (IL)-17A, IL-8, and human beta-defensin 2 (HBD)-2. Data were analyzed using analysis of variance test and Tukey honest significant differences test.

**Results:**

The expression of IL-17A, IL-8, and HBD-2 was significantly increased (p=0.000) after green tea extract administration in 7 days, whereas in 7 days, the expression of IL-8, IL-17A, and HBD-2 after EGCG and EGC administration did not give a significant result (p>0.005).

**Conclusion:**

Within the limits of this study, green tea extract has the ability as an immunomodulatory agent in an immunocompromised patient infected by *C. albicans* through expression augmentation of IL-8, IL-17A, and HBD-2 compared to EGCG and EGC.

## Introduction

The immune system is an essential mechanism to against microorganism and its toxins. The defect in the immune system could lead to an immunocompromised condition that put patients at high risk of various infections of fungal, viral, and bacterial infections [1]. One of the most common infection is fungal infection, oral candidiasis, which could lead to systemic candidiasis with a mortality rate of 60% [1,2]. *Candida albicans* is part of the indigenous microbial flora in humans and can be found in the oral cavity, and is unique among opportunistic pathogens because it is part of the normal microbial flora of the host [1,3]. *C. albicans* has been shown to play an important role in oral candidiasis, denture stomatitis, and severe periodontitis. In immunocompromised patients, mucosal immunity is not able to eliminate the adhesion of *C. albicans*. The prevalence of oral candidiasis tends to increase along with immunocompromised patients, such as human immunodeficiency virus (HIV), diabetes mellitus, iatrogenic infections, and cancer [4]. The condition causes impaired phagocytosis effect of polymorphonuclear cells, and macrophages decrease the quality and quantity of cytokines [5]. *C. albicans* infection triggers differentiation of T helper (Th) cell into Th17 to produce Interleukin 17 (IL-17) through activation IL-23 that was produced by dendritic cells. IL-17A plays an important role in mobilization and fungicidal activity of neutrophil [5,6]. In addition, activation of IL-17A through receptor IL-17R in epithelial induce the release of human beta-defensin 2 (HBD-2). HBD-2 is part of innate immunity system that plays a role in recruitment and activation of neutrophil to phagocyte against *C. albicans* [7].

The resistance to antifungal agents tends to increase nowadays and causes failure of treatment. Therefore, innovation is needed to enhance the effectivity of antifungal agents. The common anti-fungal agents gradually become more unaffordable for some Indonesians. Therefore, it is required to seek a solution to treat oral candidiasis in immunocompromised patients properly.

Current researches which are supported by Ministry of Technology and Higher Education tend to use more natural ingredients. Herbal based medicines could be the proper solution to treat some diseases. In addition, herbal medicines are abundantly provided in nature, affordable, have a low toxicity level, and are recognized safe by Food and Drug Administration (FDA) [8]. Catechin that contained in green tea could have higher bioavailability when absorbed orally.

One of the natural ingredients that are widely consumed by Indonesians is *Camellia sinensis* (green tea), which is known to act as a potent antioxidant. It contains catechins that consist of epicatechin (EC), epigallocatechin (EGC), EC gallate, and EGC gallate (EGCG). It is reported that catechin in green tea has the ability as antioxidant, anticancer, antifungal, and antivirus [9]. EGCG and EGC act as an immunomodulator by influencing the proliferation of lymphocyte T and cytokines production. Green tea extract enhances lymphoblast to induce the production of lymphocytes, while EGCG stimulates the production of IL-1α, IL-1β, monocytes, and lymphocytes [10].

The EGCG and EGC administered orally could suppress inflammation, inhibit proliferation and pro-inflammation cytokines, as well as, inhibit the activation of NF-κB [11]. This condition leads to lower level of IL-8 which plays as chemoattractant and neutrophil recruitment. The active component is responsible for biological effects such as immunomodulator, anti-tumor activity, and antimicrobial [12].

It is possible that green tea possesses immunomodulatory effect against oral candidiasis in immunocompromised patients. However, further investigation is needed to study the immunomodulatory effect of green tea extract, EGCG, EGC through increasing expression of IL-8, IL-17A, and HBD-2. This aim of this study was to develop natural based immunomodulator and anti-fungal agent to treat fungal infection in immunocompromised patients.

## Materials and Methods

### Ethical approval

This study has been approved by the Commission of Ethical Clearance, Faculty of Dental Medicine, Universitas Airlangga, Surabaya, Indonesia (No. 306/HRECC.FODM/XXI/2017).

### Animals

The research was post-test only control group design using Wistar rats as an animal model. The criteria of rats were healthy, 3 months old, and weighing around 250 g. This study using 35 immunocompromised Wistar rats induced with *C. albicans*, which are divided into 7 groups (n=5): Control group (C+); treated with green tea extract concentration 1.25% for 4 (GT 4) and 7 (GT 7) days; EGCG 1% for 4 (EGCG 4) and 7 (EGCG 7) days; and treated EGC 1% for 4 (EGC 4) and 7 (EGC 7) days.

### Experimental design

This study was conducted in Integrated Research and Testing Laboratory Biochemistry Faculty of Medicine, Universitas Airlangga. Immunocompromised condition was induced by Dexamethasone 0.8 mg/kg and Tetracycline 12 mg/kg intraperitoneally for 7 days. Blood serums were collected on day 1 and 7 to measure the immunocompromised condition through a blood test. The immunocompromised rats were then induced with *C. albicans*, by inoculating 0.1 mL saline suspension which contains 3×10^8^ *C. albicans* to tongue tissue using cotton rolls in all groups for 6 times in day 7-12. Treatment groups were treated with green tea extract, EGCG and EGC until 16 and 19 days, respectively, based on its group treatment.

### Preparation of green tea extract

Two and half gram of dried green tea leaf was mixed into 200 mL of boiling distilled water for 30 min. After being rested and filtered, the extract was freeze-dried to preserve the component substances green tea extract with a concentration of 1.25% was made by mixed 12.5 mg of dried green tea extract with 100 mL of distilled water for 30 min. It was followed by filtering the extract.

### Preparation of EGCG and EGC extract

The EGC and EGC powder was obtained from Xi’an Rongsheng Biotechnology Co., Ltd, Xi’an, PRC. There were 2.5 g of EGCG and EGC powder in 250 mL sterilized distilled water needed to make 1% EGCG and EGC solution. The solution was double filtered 30 min after mixture to gain clear EGCG and EGC solution.

### Immunohistochemistry assay (IHC)

The rats were decapitated using a combination of Ketamine and Xylazine (100 mg/kg and 10 mg kg/BB) intramuscular injection. C+ group was terminated on day 13; while GT 4, EGC 4, and EGCG 4 groups were terminated on day 16; and green tea 7, EGCG 7, and EGC 7 were terminated on day 19. Tongue tissues were collected and analyzed using IHC staining using monoclonal antibody of IL-17A, IL-8, and HBD-2 from Santa Cruz Biotech (Santa Cruz Biotechnology Inc., Dallas, Texas, USA) and examined under Olympus digital microscope (Olympus Co., Tokyo, Japan) at 400× magnification.

### Statistical analysis

The data were analyzed using SPSS software version 10.05 (SPSS Inc., Chicago, USA) with ANOVA and *post* *hoc* Tukey honest significant differences (HSD) test.

## Results

**Figure-1:**
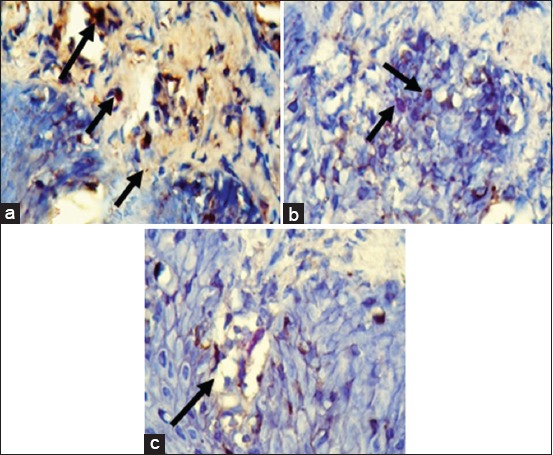
Immunohistochemistry of interleukin-8 expression (black arrow) in tongue tissue (400×) in the group which treated with green tea extract (a), epigallocatechin (EGC) gallate (b) and EGC (c) on day 7.

**Figure-2:**
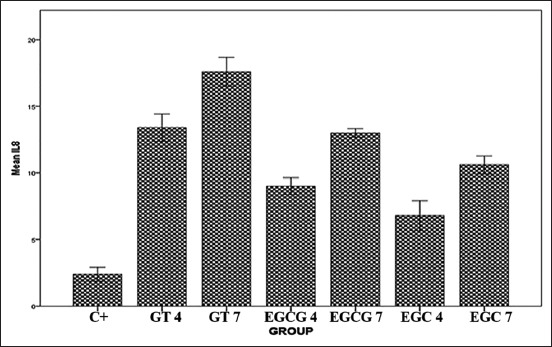
The mean of interleukin-8 expression level after treated with green tea extract, epigallocatechin (EGC) gallate, and EGC.

The IHC results of IL-8 expression are showed in Figure-1. Mean of each group for IL-8 expression was shown in Figure-2. ANOVA results showed that the expression of IL-8 was significantly different for each group (p=0.000). Tukey HSD test results in 4 days showed there was significant difference expression between GT 4 group compared to EGCG4 (p=0.011) and EGC4 (p=0.000) groups. However, there was no significant difference of IL-8 expression between EGCG 4 and EGC 4 groups (p=0.495). The expression of IL-8 in day 7 was higher than in day 4. Tukey HSD test result showed greater expression of IL-8 in GT 7 group compared to EGCG 7(p=0.000) and EGC 7 (p=0.000) groups. The expression of IL-8 between EGCG 7 and EGC 7 groups did not show a significant difference (p=0.394).

**Figure-3:**
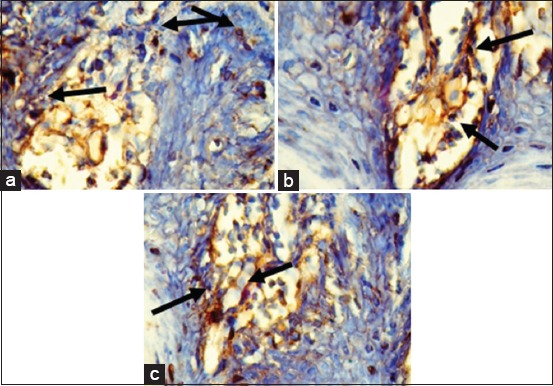
Immunohistochemistry of interleukin-17A expression (Black arrow) in tongue tissue (400×) of the group with green tea extract (a), epigallocatechin (EGC) gallate (b), EGC (c) on day 7.

**Figure-4:**
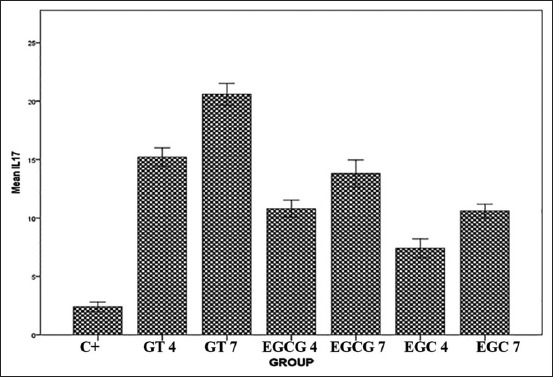
Interleukin-17A expression level after treated with green tea extract, epigallocatechin (EGC) gallate, and EGC.

The expression of IL-17A is shown in Figure-3, and the mean is shown in Figure-4. The expression of IL-17A was significantly different in each group (p=0.000). Tukey HSD test results in 4 days showed there was a significant difference between GT 4 group compared to EGCG 4 (p=0.010) and EGC4 (p=0.000) groups. Meanwhile, the comparison of IL-17A expression between EGCG 4 and EGC 4 did not show a significant difference (p=0.076). The expression of IL-17A on day 7 is higher than on day 4. HSD test result showed higher expression of IL-17A in GT 7 group compared to EGCG 7 (p=0.000) and EGC 7 (p=0.000) groups. The expression of IL-17A between EGCG 7 and EGC 7 groups did not show a significant difference (p=0.110).

**Figure-5:**
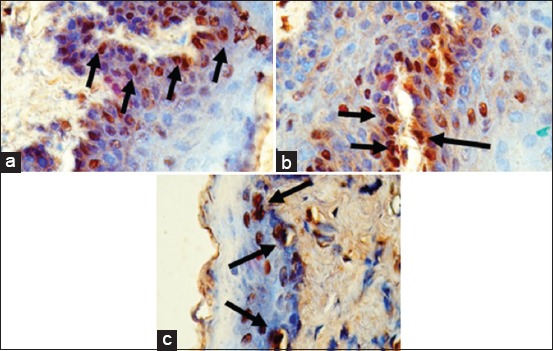
Immunohistochemistry of human beta-defensin-2 expression (black arrow) (400×) of the group treated with green tea extract (a), epigallocatechin (EGC) gallate (b), EGC (c) on day 7.

**Figure-6:**
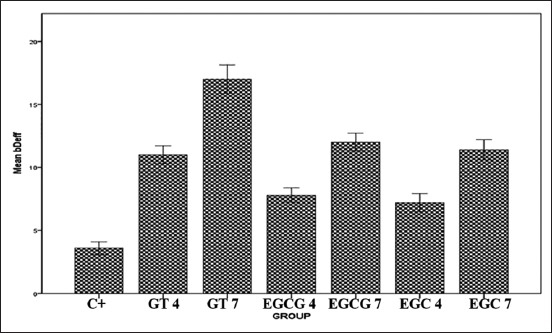
Human beta-defensin-2 expression level after treated with green tea extract, epigallocatechin (EGC) gallate, and EGC.

The expression of HBD-2 is shown in Figure-5, while the means are shown in Figure-6. The expression of HBD-2 was significantly different for each group (p=0.000. Tukey HSD test result showed no significant difference in HBD-2 expression in GT 4 group compared to EGCG 4 group (p=0.080) but showed a significant difference if compared to EGC 4 group (p=0.023). The expression of HBD-2 between EGCG 4 and EGC4 groups did not show any significant difference (p=0.998). The expressions of HBD-2 in day 7 are greater than on day 4. Tukey HSD test result showed that the expression of HBD-2 in GT 7 group was significantly different compared to EGCG 7 (p=0.000) and EGC 7 (p=0.001) groups. Meanwhile, the expression of HBD-2 between EGCG 7 and EGC 7 groups did not show a significant difference (p=0.998).

## Discussion

The current management of oral candidiasis is not effective enough, especially for immunocompromised individuals, due to high risk of recurrence. Therefore, adjunctive therapy is needed to get a better result. Recently, approximately 80% of antimicrobial, cardiovascular, immunosuppressive, and anticancer drugs are of plant origin. It is widely accepted that more than 80% of drug substances are either directly derived from natural products or developed from a natural compound [13]. One of the plants that have a therapeutic effect is green tea (Camellia Sinensis). Green tea extract is known to have anti-oxidant and antifungal properties [14].

Prevalence of oral candidiasis tends to increase each year as increasing number of the immunocompromised patient due to diabetes mellitus, prolonged consumption immunosuppressant and antibiotic, transplant organ recipient, cancer survivor, and patient with HIV/AIDS [15]. Prolonged and irrational usage of anti-fungal (azole) is believed to contribute to the higher prevalence of oral candidiasis, which leads to treatment failure [16]. Therefore, innovation to enhance anti-fungal effectivity is needed. In immunocompetent patients, every microorganism including fungi is controlled by the immune response, whether specific or non-specific. The immune system of immunocompromised patient fails to eliminate the adhesion of Candida that leads to oral candidiasis [6].

Several antifungal agents, nystatin, and azole are reported to be resistant to oral candidiasis. Meanwhile, improper treatment of oral candidiasis may lead to systemic infection of Candida that increases mortality rate to 60% [17]. Green tea extract which contains EGCG and EGC is considered as an herbal-based therapy for oral candidiasis. Green tea extract is generally recognized as safe by the FDA Safety of the USA [8], because of green tea that is traditionally consumed as food relies on that history of food use as evidence of safety.

This study showed that IL-8 is increased on day 4 and 7 after treated green tea extract. Green tea extract showed higher IL-8 expression compare to EGCG and EGC due to green tea extract contains various active components which synergistically increase IL-8 expression. IL-8 is important due to chemo-attractant properties which recruit neutrophils for phagocytosis to inhibit *C. albicans* colony formation. A previous study by Steubesand *et al*. [18] showed oral administration of EGCG and EGC could inhibit the synthesis of pro-inflammation cytokines and the activation of NF-κB which leads to decreasing IL-8 expression and increasing HBD-2. Various components contain in green tea extract work synergistically to enhance the expression of IL-17A and IL-8 which play as a chemoattractant to release HBD-2.

The expression of IL-17A is shown in Figure-4 after treated with green tea extract was significantly different in each group (p=0.000). Meanwhile, the comparison of IL-17A expression between the group treated with EGCG and EGC did not show a significant difference (p=0.076) in day-7 is higher than on day-4. There is the IL-17A play vital role in mobilization and activity of neutrophils against infection, and it will be expressed by the activity of Th17 through RORγt and increases the number of neutrophils to inhibit oral candidiasis, even in immunocompromised patients [19,20]. Green tea extract contains flavonoids which have an immunomodulatory effect, thus enhance IL-2 production which activates the proliferation and differentiation of T cells differentiate into Th1 and Th2, Th1 cells secrete various cytokines Interferon gamma (IFN-γ) to activate macrophages [21]. Flavonoid contained in green tea extract enhances proliferation of lymphocytes could affect CD4+ cells to activate Th17 and produces IL-17A [22]. It is followed by epithelial induction through IL-17R, which activates the synthesis of HBD-2, IL-8, and G-CSF. Deficiency of IL-17A led to a neutrophil deficiency which increases the risk of systemic *C. albicans* infection.

The non-specific immune response is triggered as α-mannans binds to *C. albicans* cell wall. The binding stimulates specific immune response through Th17. The activity of Th17 cells through inducing Treg will reduce expression of Dectin-2 in *C. albicans* [23]. These conditions stimulate the expression of various markers such as RORγt, IL-17A, and HBD-2, as well as the number of neutrophils. A sufficient number of *C. albicans* might be increased and initiate oral candidiasis.

Green tea extract, EGC, and EGCG inhibit Candida biofilm formation and preformed biofilm. Higher EGCG concentration is reported to be substantial for *in vivo* activity of *C. albicans* proteasome chymotrypsin-like. Proteasome activities disturb cellular metabolism and yeast structure [15]. In *C. albicans* infection, macrophages release various pro-inflammation cytokines supported by IL-23 such as IL-17A, IL-21, and IL-22, and would trigger differentiation of Th into Th1, Th2, and Th17. IL-17A is the most prominent cytokines released by Th17 that was activated by IL-23. It is beneficial to trigger epithelial to secrete antimicrobial peptides and recruit neutrophils for phagocytosis, *C. albicans* in particular [10,12]. In certain condition, there would be declined secretion of IL-17 due to such mechanism by *C. albicans* to avoid the host immune response.

HBD-2 expressions are higher in green tea extract group compared to EGC and EGCG. Higher expression HBD-2 in green tea extract group due to its ability to activate HBD-2 release as effector molecules in oral mucosal innate immunity. HBD-2 is part of mucosal primary defense mechanism against *C. albicans* through several mechanisms in the cell membrane, such as binding ions Ca2+ and Mg2+ [24]; consequently, there would be ion and peptide exchange that interfere the stability of *C. albicans* membrane. Instability of membrane leakage would lead to apoptosis of *C. albicans*. This shows that various components contained in green tea extract work synergistically and simultaneously to increase IL-8 and IL-17A expressions. Lack of HBD-2 leads to reduce recruitment and activation of neutrophils and affects its phagocytic ability against *C. albicans*. Miramón *et al*. [25] stated that oral candidiasis can be cured in immunocompromised individuals. Decreasing number of HBD-2 stimulates reduce the activity and recruitment of neutrophil moreover lower phagocytosis activity against *C. albicans*.

## Conclusion

The expressions of IL-17A, IL-8, and HBD-2 are higher after treated with green tea extract, compared to treatment with EGC and EGCG. The results show that green tea extract gives better immunomodulatory effect, compared to EGC and EGCG. It is concluded that green tea extracts possess immunomodulatory effect against *C. albicans* infection in immunocompromised patients by increasing the expression of IL-8, IL-17A, and HBD-2.

## Authors’ Contributions

All authors participated equally in the study plan and design. RAP, UK, and RPDI collected the samples from the farms and prepared the animal models. RPR and DAP assisted in the laboratory work. MR carried out the statistical analysis of data and reporting the results. All authors collaborated in writing, revising, and improvement of the article for publication.
